# 3D printing individualized heel cup for improving the self-reported pain of plantar fasciitis

**DOI:** 10.1186/s12967-018-1547-y

**Published:** 2018-06-18

**Authors:** Lan Li, Longfei Yang, Fei Yu, Jianping Shi, Liya Zhu, Xianfeng Yang, Huajian Teng, Xingsong Wang, Qing Jiang

**Affiliations:** 1 School of Mechanical Engineering, Southeast University, No. 2 Southeast University Road, Nanjing, China; 2 Department of Sports Medicine and Adult Reconstructive Surgery, Drum Tower Hospital Affiliated to Medical School of Nanjing University, Nanjing, China; 3 Drum Tower of Clinical Medicine, Nanjing Medical University, Nanjing, China; 4 School of Electrical and Automation Engineering, Nanjing Normal University, Nanjing, China; 5 Model Animal Research Center, Nanjing University, Nanjing, China; 6 Institute of Medical 3D Printing, Nanjing University, Nanjing, China

**Keywords:** Heel cup, Heel pain, Finite element, 3D printing, Relief pain, 3D scanning

## Abstract

**Background:**

To explore the therapeutic effect and the biomechanical mechanism of 3D printing individualized heel cup in treating of plantar heel pain.

**Methods:**

The clinical effect was evaluated by plantar pressure analysis and pain assessment in participants. Its biomechanical mechanism of protecting the plantar heel was explored using finite element simulation.

**Results:**

The individualized heel cup could support and protect the osseous structure and soft tissue of plantar heel while walking and jogging, as well as significantly reduce the self-reported pain after being worn for 4 weeks. The nylon heel cup could alter the load concentration of the heel as well as decrease the load affected on plantar fascia and calcaneus bone. It also provided an obvious support for heel pad.

**Conclusion:**

To summarize, the 3D printed individualized heel cup can be used as an effective method for the treatment of plantar heel pain.

## Background

Plantar heel pain (PHP), also known as plantar fasciitis, jogger’s heel, tennis heel, and policeman’s heel [1], is a painful syndrome that occurs around the calcaneus. Although the term itself might be vague, the chronic pain that originates at the plantar fascia following weight bearing is the unfortunate reality of many. The pain, which could lead to a reduction in sporting and everyday activities, has been described as burning, aching, and occasionally lancinating [2]. It is the most common foot musculoskeletal disorder encountered by health professionals, that has a negative impact on health-related quality of life in those who experienced it [3, 4]. This condition occurs in approximately 2 million Americans per year, while the prevalence rates in UK and Australia are 4.6 and 17.4%, respectively [5–7]. Besides athletic and non-athletic populations, it has also observed in the sedentary population [8–10]. It isn’t gender-specific, and while it generally affects the mid-aged; it has also been observed in children and elderly [9, 11]. Despite the high prevalence of PHP, its real etiology is poorly understood. Previous studies have suggested that it is probably multifactorial, and related with plantar fasciitis, heel spur, calcaneal apophysitis, bursitis, posterior calcaneal exostosis, local bruises, Achilles tendonitis, and trapped nerve [12, 13]. The repetitive micro-trauma associated with persistent load-bearing caused by excessive stress at the calcaneal attachment of the plantar fascia has shown to be a major risk factor for heel pain [14].

The initial treatments for PHP include padding, strapping of the foot, orthotic insoles, oral anti-inflammatories, and a local corticosteroid injection [15–19]. If the therapy program is effective, patients usually have a positive response within 6 weeks of initiation treatment. The second line treatment includes orthotic devices, night splints, repeat corticosteroid or botulinum toxin injections, a course of physical therapy, and cast immobilization during activity [15, 18, 20–25]. For most patients, clinical response after this line of treatment usually occurs within 2–3 months [26–28]. If the symptoms are still present within 1 year after the first and/or second line treatment, the patients should face the final line of treatment, which usually includes plantar fasciotomy, heel spur resection, nerve release, and other kinds of surgeries [12, 29]. However, simple removal of the heel spur does not seem to promote the successful outcome in the surgical treatment in most cases, i.e. the combination of plantar fasciotomy and nerve release appear to be necessary [30, 31]. In sum, the three lines of treatment are gradually complementing, where the last two lines of treatment, and especially the surgical treatment, are avoidable if the first line of treatment is successful. The decision to include analgesia or anti-inflammatory medication in line 1 and line 2 treatment is considered only where its use has been shown to detrimental or to have an off-label effect [32]. The key factor for successful rehabilitation in the early period is altering the kinetic chain of the lower limb [33, 34].

Heel cup, or foot orthosis, is the most common rehabilitation method used for the initiation treatment. The traditional fabrication process of orthosis includes plastics injection molding and plaster molding. The fabricated products are usually non-individualized, heavy, plump and uncomfortable to wear [35], and compared with individualized heel cups, the pre-fabricated products are not very effective in some cases [36]. In order to overcome these shortcomings of traditional orthosis, additive manufacturing (AM) has been introduced to the field. The new technology, also known as rapid prototyping (RP) or 3D printing, has unique advantages in medical application. It allows the fabrication of complex 3D physical objects from large sizes to microstructures in a wide range of materials, like plastics, metals, nylons, and biocompatible ones [37–42]. Furthermore, it is possible to acquire the digital models of the human body by combining the 3D scanning technology and rebuilding or repairing surface anatomy by 3D printing [43]. Based on this premise, we designed and tested the therapeutic effect of individualized heel cup fabricated using 3D printing. The digital models of heel cup were designed to closely fit the surface of human feet and were tested on 16 participants with plantar heel pain who were enrolled in the study. Additionally, the functionary mechanism of 3D printing heel cup was explored by finite element (FE) simulation.

## Methods

The experiment was divided into three parts: heel cup design and fabrication, clinical evaluation and FE model simulation. All methods in this study were carried out in accordance with relevant guidelines and regulations. All experimental protocols in this study were approved by the committee of Drum Tower Hospital affiliated to Medical School of Nanjing University.

### Heel cup design and fabrication

**Fig. 1:**
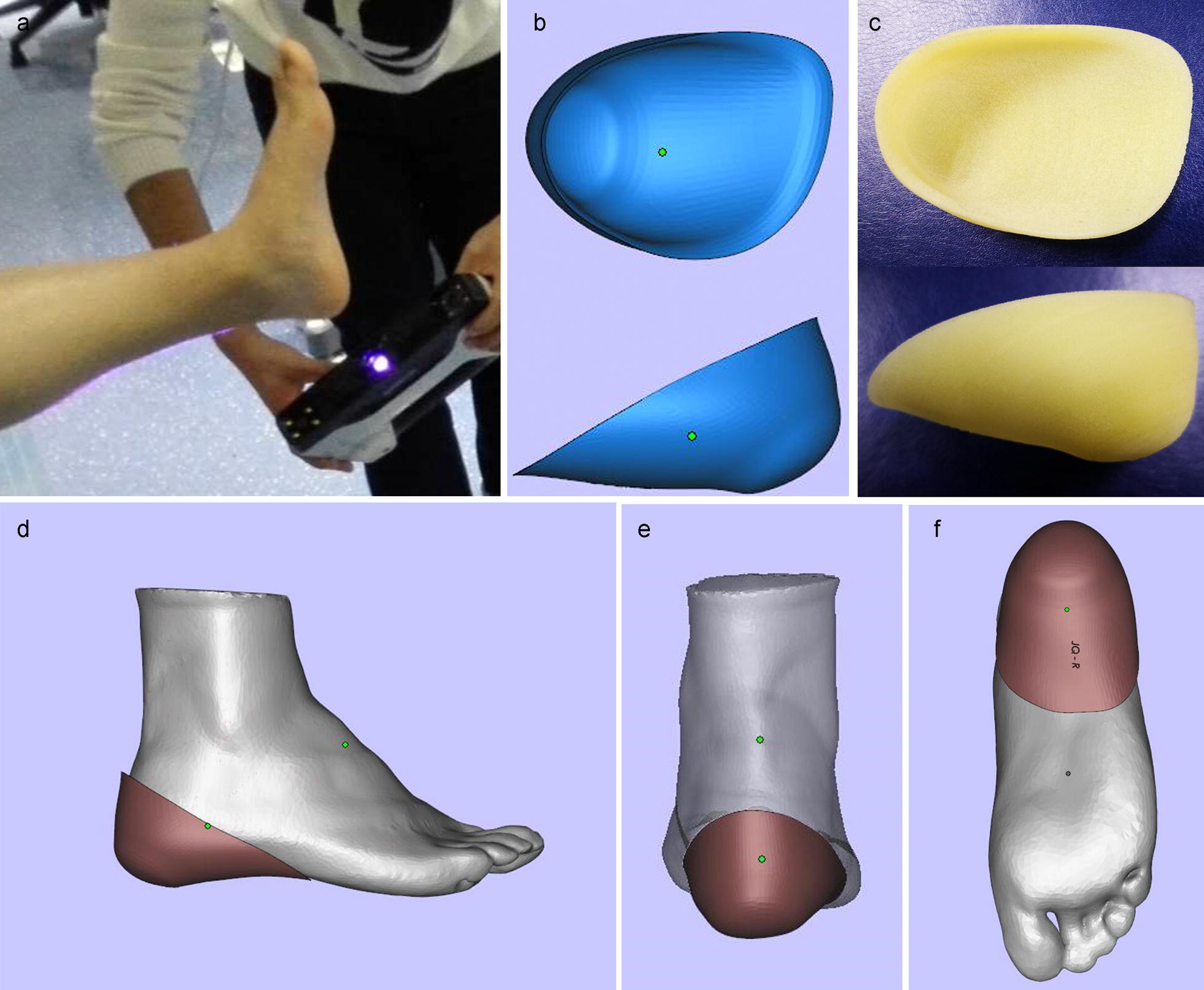
3D scanning process and the design of individualized heel cup. **a** 3D scanning process. **b** The shape of heel cup. **c** The finished product made by 3D printing. The 3D impression drawing after wearing the individualized heel cup, the lateral view (**d**), the rear view (**e**), and the bottom view (**f**)

Patients’ feet and calves were scanned by a 3D handheld scanner (EinScan-Pro, Shining 3D, China) in a high-definition mode (Fig. 1a), and scans were exported in STL file format for post-processing. The internal surface of heel cup was designed in Magics 21.0 (Materialise, Belgium) by a Boolean Operation with plantar heel. The heel cup was 5 mm thick and it completely wrapped the plantar heel from the upper margin of the calcaneus to the arch of the foot (Fig. 1b, c, e, f). The digital modes of heel cups were imported in an EOS P110 nylon 3D printer (Electro Optical Systems, Germany) and fabricated by selective laser sintering (SLS) rapid prototyping technology. The diameter of the spot was 0.05 mm, the thickness of the powder layer was 0.03 mm, and the power of laser was 2.0 kW. The 3D printed plantar heel cup was shown in Fig. 1c.

### Clinical evaluation

#### Plantar pressure analysis

The plantar pressure analysis was performed by a Gait analysis machine (Zebris, Germany). We measured the stress characteristics of plantar surface under the walking and jogging stances, with and without heel cups, respectively. Each operation was repeated three times so as to verify the accuracy of measurement.

#### Participants

A total of 16 participants (6 females and 10 males) were recruited among patients at Drum Tower Hospital affiliated to Medical School of Nanjing University and among students at Nanjing University. A diagnosis of unilateral or bilateral heel pain associated with plantar fasciitis was made by an experienced sports medicine doctor. It was not known whether any of patients had heel spurs coexisting with heel pain due to the absence of radiograph results. The length of time that a patient experienced pain was from 1 to several years, while all the subjects reported increased pain during activity. Some of them had tried other treatments, like over-the-counter heel pad, oral painkillers, and anti-inflammatories. None of these treatments significantly reduced the pain.

#### Assessment protocol

In the present study, we used a Likert-type scale, divided into 10 levels, from 1 point (no pain) to 10 point (extreme pain), to evaluate the intensity of pain. Subjects were asked to rate their pain at the initial visit and after wearing 3D printed heel cups for 4 weeks. The statistical analysis was performed with SPSS 19.0 (SPSS Inc, USA). All the data were recorded as mean ± SD and were evaluated by an unpaired Student’s *t* test. Two-tailed P value < 0.05 was considered statistically significant.

### FE model simulation

#### Computed tomography (CT) scanning and 3D reconstruction

A CT was performed with a GE Lightspeed 16 CT equipment on a 29 years old male weighing 74 kg. The scan was realized for both feet at the neutral posture with a slice distance of 0.625 mm and a field of view (FOV) of 500 mm.

The 3D models of bone structure and soft tissues were reconstructed by MIMICS 19.0 (Materialise, Belgium). The DICOM image files were imported in the software and segmented on the basis of gray intensities. The separated 3D reconstruction for each bone was accomplished with CT Bone Segmentation operation. The structure of soft tissues was obtained by a Boolean Operation between the whole structure and the bone. The tendon and ligament structures were not specifically accounted in the 3D reconstruction, but they were all in the constructer of soft tissue. All the parts were exported as STL files, respectively for future 3D computer aided design (CAD) operations and FE simulation.

#### The load and boundary constrains

A vertical force corresponding to half of the body weight was simulated and applied at the ground support reference point. The effect of Achilles tendon loading in the standing foot was approximately 75% according to Cheung’s study [44]. For a participant with body mass of 75 kg, a pure vertical compression load defined by a vertical force (375 N) was applied as the ground reference point. We just considered the gastrocnemius muscle force on the Achilles tendon and ignored the effects of the other internal and external muscle forces. Bone segments were assumed frictionless, and the ground support and the plantar surface were defined with frictional contact method, and a coefficient of friction of 0.6. The surfaces of the soft tissues, tibia, and fibula were fixed through analysis time via kinematic constrain, and allowed uniquely the plate movement in the vertical direction.

#### FE simulation

The STL files from 3D reconstruction were imported in Geomagic Studio 12.0 (Geomagic, USA) to optimize meshes, and to reconstruct surfaces for finite element analysis; the meshing was operated by HyperMesh 2017 (Altair, USA). The finished models were imported and assembled in the Abaqus 2017 (Dassault, France). All the bone segments were considered isotropic and liner-elastic, and no distinction was found between cortical bone and cancellous bone. The bone material behavior was linear with a Young’s modulus as of 7300 MPa and a Poisson’s ratio of 0.3. The soft tissue was considered non-linear and hyperelastic [45, 46]. The heel cup was made out of nylon, and the material behavior was linear with a Young’s modulus of 1700 MPa and a Poisson’s ratio of 0.34. Based on these parameters, we sed the software to simulate the quiet standing stance (weight load) with and without the heel cup.

## Results

### Clinical evaluation

**Fig. 2:**
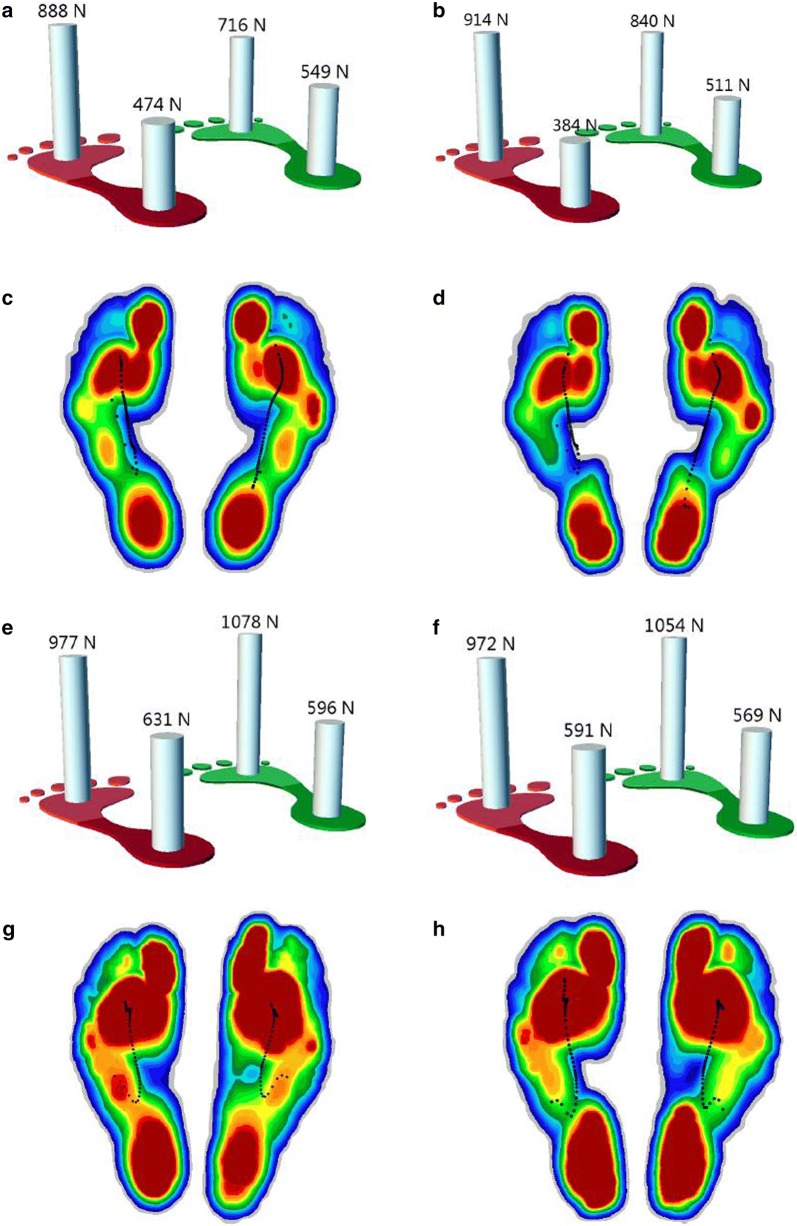
Plantar pressure analysis during walking (**a**–**d**) and jogging (**e**–**h**). **a**, **e** Overall load of forefoot and hind foot with bare foot. **b**, **f** Overall load of forefoot and hind with heel cup. The load increased on forefoot and decreased on hind foot. **c**, **g** Stress nephogram of plantar tissue with bare foot. **d**, **h** Stress nephogram of plantar tissue with heel cup. The region with orange on mid foot was replaced by green, indicating that the load in this region was reduced. The color that changed from red to blue represented the stress variation from large to small

The plantar stress measured by the gait analysis machine is shown in Fig. 2. Briefly, the load on both mid-foot and hind foot in two postures decreased after wearing the heel cup. The applied force to the forefoot increased during walking and decreased during jogging after wearing the heel cup.

**Fig. 3:**
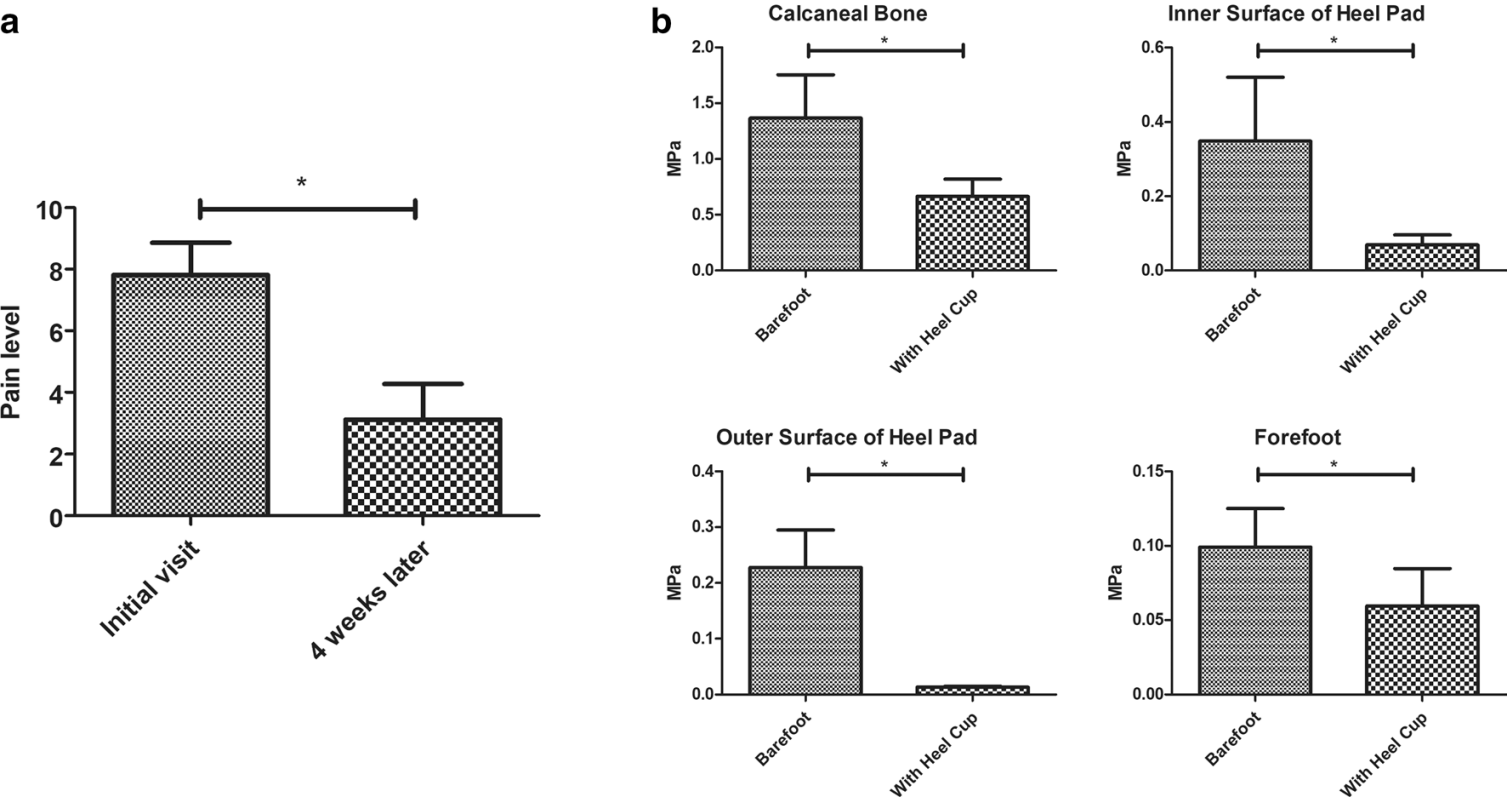
**a** The pain score of participants. **b** Statistical results of stress in each region of foot. Values were mean ± SD. *P < 0.05

The age of participants ranged from 8 to 58 years, and the average body weight was 64.2 ± 19.95 kg. Nine subjects were diagnosed with bilateral plantar fasciitis. After wearing heel cups for 4 weeks, all the participants reported the reduced pain. Only 2 of them reported a slightly reduced level as the other subjects’ pain was all significantly relieved. What’s more, all the participants reported longer activity time with the heel cups. The pain score was 7.8125 ± 1.0468 at the initial visit, decreasing to 3.125 ± 1.1475 after 4 weeks (Fig. 3a).

### FE model validation

**Fig. 4:**
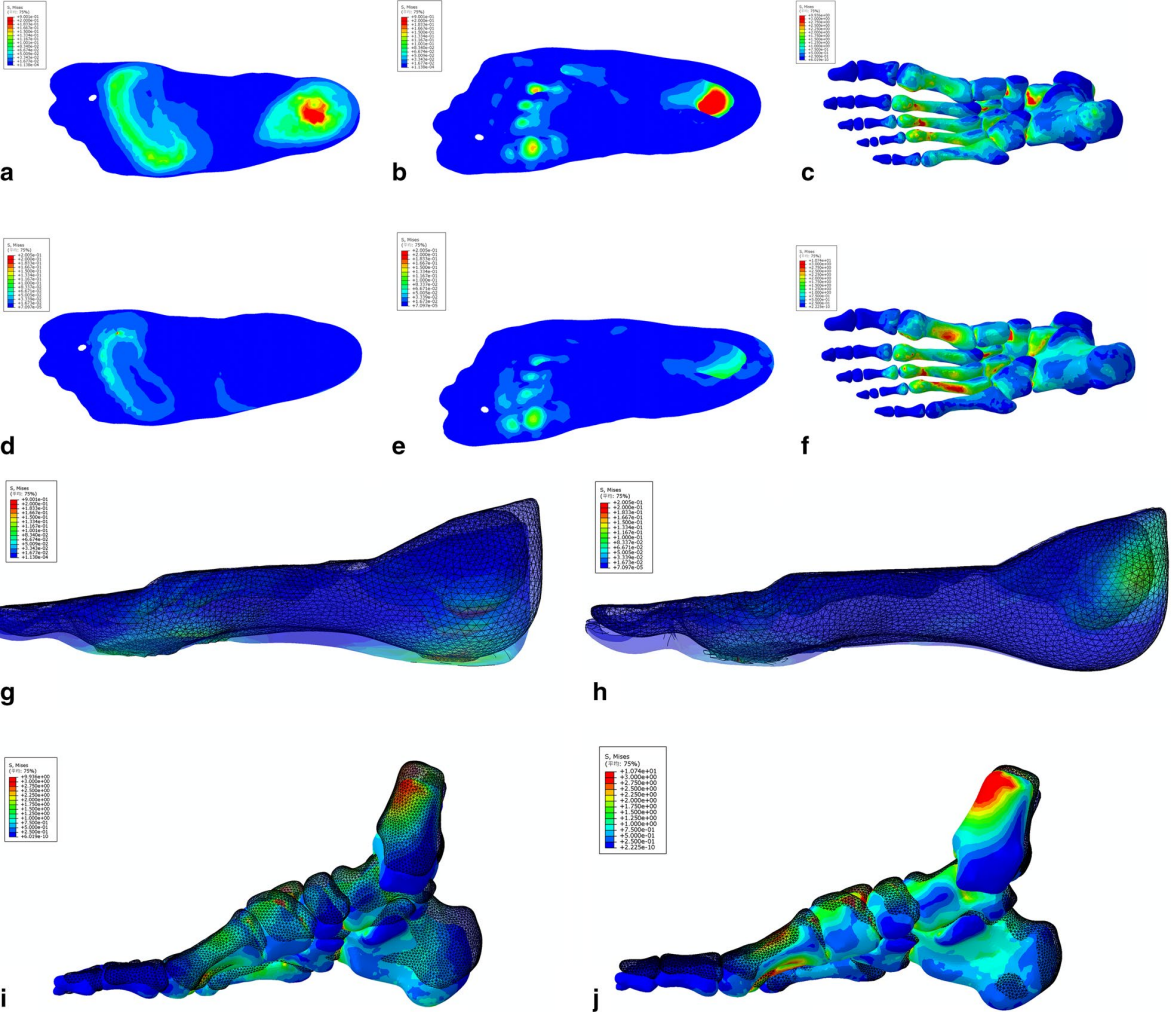
The plantar pressure distribution (**a**–**f**) and the structure behavior (**g**–**j**) calculated by FE simulation. The bottom view (**a**) and top view (**b**) of plantar tissue, and the bottom view of ossature (**c**) with bare foot demonstrated that the load applied on plantar heel and forefoot was higher than other regions. The bottom view (**d**) and top view (**e**) of plantar tissue, and the bottom view of ossature (**f**) with heel cup showed that the red region in plantar heel disappeared and the color of calcaneus bone transferred from green to blue, indicating that the load decreased notably. The color that changed from red to blue represented the stress variation from large to small. The deformation of plantar soft tissue with bare foot (**g**) was more obvious than the tissue covered with heel cup (**h**). The same phenomenon could be observed on ossature structure between bare foot group (**i**) and heel cup group (**j**). The pattern made up of meshes represented the original shape, and the entity pattern represented the shape under compression

In general, the load transmission between ground support and plantar surface was defined by the introduction of contact pairs; large deformations and non-linear geometrical analysis associated with material nonlinearities were also considered. The von Mises stress in the plantar soft tissue and bone were shown in Fig. 4. The loads on the calcaneus bone, the fat pad, the plantar, and the forefoot obviously decreased according to the color variation on the stress nephogram (Fig. 3b). All the elements in the stress concentration area were selected and analyzed to achieve the average value of the load. The results were shown in Table 1. Besides the decrease in the load, the compression rate of foot fat pad also decreased from 49.4 to 28.7%; the simulation results were shown in Fig. 4. Furthermore, the angle of interior and lateral longitudinal arch also decreased to a certain extent (Table 2).

**Table 1:** Results of load variation on different regions of foot

Region	Barefoot (MPa)	With heel cup (MPa)
Calcaneal bone	1.3657 ± 0.3907	0.7089 ± 0.1319
Inner surface of heel pad	0.3387 ± 0.1787	0.0688 ± 0.0271
Outer surface of heel pad	0.2278 ± 0.0672	0.0129 ± 0.0016
Forefoot	0.0928 ± 0.0194	0.0596 ± 0.0251

**Table 2:** Results of foot arch angle variation

	Original	Barefoot	With heel cup
Interior longitudinal arch	112.831°	114.852°	113.660°
Lateral longitudinal arch	152.348°	153.710°	153.386°

## Discussion

The results of the present study demonstrated that the individualized heel cup could alter the dynamics of the heel, and could significantly improve the self-reported pain caused by the plantar heel pain. Almost all of the participants reported a definite alleviation of heel pain, while FE simulation revealed the causation of this phenomenon. The biomechanical abnormalities and mechanical overload could lead to the excessive tensile strain, responsible for the plantar fasciitics [47]. What’s more, in order to resist arch elongation, the tensile loads of the plantar fascia increase during the stance and the heel rising stage [48]. The plantar fascia is very susceptible to injury under long and repeating overload, which may shorten its effective length and initiate the development of plantar fasciitics. According to the FE simulation, the warping of heel cups obviously exerts the opposite force on the plantar soft tissue and calcaneus bone, which in turn might directly cause the heel pain. Furthermore, the plantar pressure analysis exhibited that the load on mid foot during walking and jogging also decreased, which meant the direct load from plantar fascia had also been reduced. Compared with patients who did not wear a cup, the variety of longitudinal foot arch angle was smaller after wearing the heel cup. The work of fascia to resist collapse of the arch during gait become lighter. Through this way, it could release the tensile stress of the fascia and reduce the bending angle of foot arch.

**Fig. 5:**
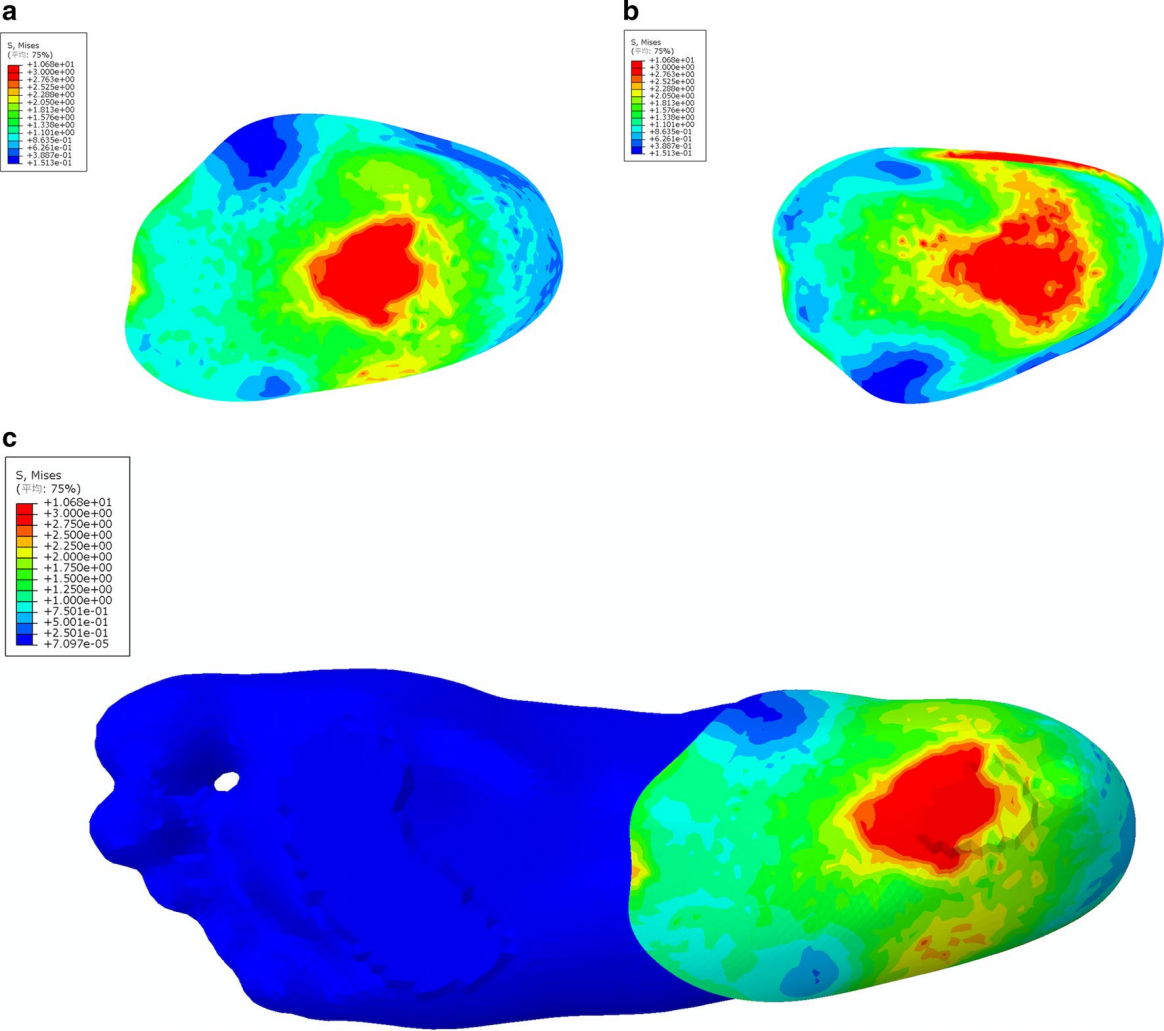
The pressure distribution of heel cup calculated by FE simulation. The bottom view (**a**), the top view (**b**) of heel cup, and the bottom view of foot (**c**) with heel cup indicated that the heel cup bore the load instead of heel pad

Besides negatively affecting the fascia through increased tension, the biomechanical factors can also disrupt energy dissipation in the heel, especially in the heel pad, which is an organized elastic adipose tissue very important for shock absorbtion [49]. Heel pad is compressed during standing, walking or running. The repetitive, cumulative fatigued micro-trauma can disrupt the balance between fatigue damage and routine repair [50]. The irreversible damage leads to an increased inflammatory, edema, and heel pad thickness. Due to these alterations, the stiffness as well as the capacity to attenuate shock, decrease. As shown in Fig. 1, the patient’s foot was 3D scanned without external pressure, while the shape of the plantar heel soft tissue was physiologic. The individualized form of heel cup was designed based on the surface morphology of patient’s plantar heel so that it can fit the skin closely. The nylon heel cup can absorb energy and buffer the shock, due to good quality material that supports elasticity and stiffness. In such manner, the heel cup can provide a good supporting role for the heel pad. The FE simulation also supported this assumption. The function of heel pad was transformed from bearing load to transfer stress, while the stress applied on the heel pad notably decreased. As shown in Fig. 5, the 3D printing heel cup bore the load; the stresses applied on the inner/outer sides were 3.6793 ± 0.819 and 4.3176 ± 1.7533 MPa, respectively.

**Fig. 6:**
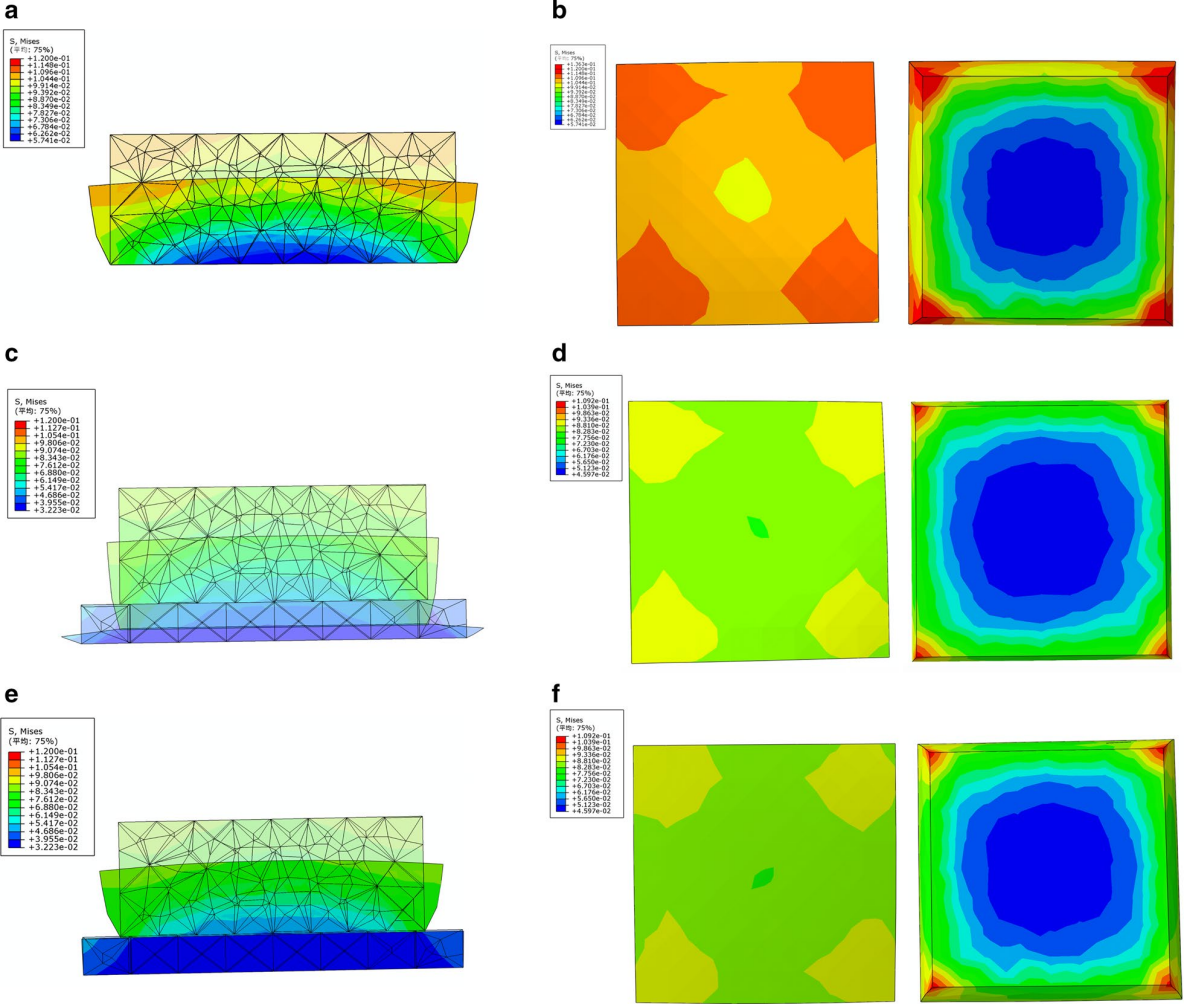
The results of the simplified FE model simulation. The three-view drawings of no-cup group (**a**, **b**), soft cup group (**c**, **d**), and hard cup group (**e**, **f**) demonstrated the deformation and stress of soft tissue and cup. The upper cube represented the soft tissue and the material parameters were same as soft tissue. The cube below represented the heel cup and the tensile modulus was set as 100 and 1800 MPa, respectively. The pattern made up of meshes represented the original shape, and the entity pattern represented the shape under compression. The color that changed from red to blue represented the stress variation from large to small

Unlike other pre-fabricated commodities, the individualized heel cup needs to be customized. The common design for commodities with a flat bottom and a low side wall could neither enwrap nor support the heel effectively. The soft tissue becomes deformed as the heel comes into contact with these insoles. The 3D printing heel cups could completely fit the patients’ individual physiological curve, thus minimizing the deformation degree of the soft tissue. In order to achieve therapeutic effect, the tensile modulus should be fitting. To verify this hypothesis, we set up a simplified model and analyzed it by FE simulation. According to the stress nephogram shown in Fig. 6, the stress applied on the soft tissue reduced if the heel cup was worn. The deformation degree was inversely proportional to the strength of the heel cup within certain range. On the other hand, the cup could absorb energy and be crushed simultaneously if the tensile modulus were too low, or if structure design was irrational. As shown in Fig. 6c, although the compression ratio and stress of soft tissue were smaller, the cup gets completely crushed with a low tensile modulus. As the strength of the cup increased (Fig. 6e), the compression ratio increased from 22 to 35% (3.3 mm vs. 5.2 mm), but had no influence on the stress from soft tissue. In our previous study, silica gel, nylon, nylon glass fiber, and photosensitive resin were tested by volunteers. The silica gel was too soft to protect the heel, while the photosensitive resin was too hard. The suitable strength, comfort, and curative effect made nylon and nylon glass fiber were the most appropriate materials for fabricating 3D printing heel cups. Nylon was finally selected as a raw material due to affordable cost, as well as short fabricating period that approximately takes about 5 h. Briefly speaking, 15 min for 3D scanning, 20 min for post-processing and designing, and 3–4 h for 3D printing.

However, there are still some limitations in this study. Due to the limited instruments, the stress nephogram of plantar pressure could not clearly show the variation in stress, but only calculate the overall load. Because of the use of FE simulation, the imaging examination methods were not employed to achieve the real time images of plantar heel under different conditions. What’s more, the number of participants was not large enough to provide strong clinical evidence for the obtained results. Thus, the main objective of our future study is to enlarge the sample size, including dividing participants in accordance with ages, symptom levels, and exercise status, and to evaluate the symptoms improvement more systematically. Moreover, the study to discuss the application potential of individualized heel cups in the athletes, especially for the runners and walkers, for the purpose of prevention and rehabilitation sports injury is in progress.

## Conclusion

In this study, we fabricated individualized heel cup using 3D printing technology. To verify its effectiveness, an in vivo experiment was conducted using a gait analysis system to measure the plantar pressure, while the clinical therapeutic effect was evaluated in some participants. Additionally, FE simulation was introduced to explore its mechanism. In conclusion, the individualized heel cup made by the combination of 3D scanning and 3D printing resulted to be an effective method for the treatment of plantar heel pain.

## Abbreviations

PHP: plantar heel pain
FE: finite element
FOV: field of view
CAD: computer aided design
